# The genome sequence of the Seraphim, *Lobophora halterata* (Hufnagel, 1767)

**DOI:** 10.12688/wellcomeopenres.18713.1

**Published:** 2022-12-23

**Authors:** Douglas Boyes, University of Oxford and Wytham Woods Genome Acquisition Lab, Darwin Tree of Life Barcoding collective, Wellcome Sanger Institute Tree of Life programme, Wellcome Sanger Institute Scientific Operations: DNA Pipelines collective, Tree of Life Core Informatics collective, Peter W.H. Holland, Darwin Tree of Life Consortium

**Affiliations:** 1 UK Centre for Ecology and Hydrology, Wallingford, Oxfordshire, UK; 2 Department of Biology, University of Oxford, Oxford, Oxfordshire, UK

**Keywords:** Lobophora halterata, the Seraphim, genome sequence, chromosomal, Lepidoptera

## Abstract

We present a genome assembly from an individual female *Lobophora halterata* (the Seraphim; Arthropoda; Insecta; Lepidoptera; Geometridae). The genome sequence is 315 megabases in span. The complete assembly is scaffolded into 32 chromosomal pseudomolecules with the Z and W sex chromosomes assembled. The mitochondrial genome has also been assembled and is 15.7 kilobases in length.

## Species taxonomy

Eukaryota; Metazoa; Ecdysozoa; Arthropoda; Hexapoda; Insecta; Pterygota; Neoptera; Endopterygota; Lepidoptera; Glossata; Ditrysia; Geometroidea; Geometridae; Larentiinae; *Lobophora; Lobophora halterata* (Hufnagel, 1767) (NCBI:txid934876).

## Background

*Lobophora halterata* (the Seraphim) is a delicately patterned moth in the family Geometridae. The adult has a series of wavy grey and brown stripes across broad white forewings, which at rest form effective camouflage against tree bark. The moth is found across central and northern Europe, with scattered records from Russia and Japan ( GBIF Secretariat, 2021). *L. halterata* has a single generation per year in the UK: larvae feed on aspen and polar, overwintering occurs as a pupa, and adults have a short flight period in May and June ( Waring & Townsend, 2017). Males of this species have a particularly unusual hindwing feature that explains the origin of the common name and the scientific name. The common name refers to a type of angel in Jewish, Christian and Islamic texts; the seraphim are generally described as six-winged angels that act as guardians of the throne of God ( Holland, 2012). The Seraphim moth does not actually have six wings, but there is a ‘concertina’ or Z-fold on the trailing edge of hind wing giving the impression of a small third pair of wings lying on top of the hindwings. There is also error in entomological etymology: in religious texts seraphim is the plural of seraph, whereas in the moth the plural term has become singular ( Holland, 2012). The genus name *Lobophora* refers to this ‘lobe’ of wing tissue, as does the specific name *halterata* which draws comparison to the lobe-like halteres of Diptera ( Maitland Emmet, 1991). The function of the unusual hindwing lobe of *L. halterata* is unknown, although the fact it is restricted to males suggests it likely to have a sex-specific role, potentially associated with a scent organ ( Hobby, 2009). The developmental genetic basis of the morphological feature is entirely unknown.

The genome of *L. halterata* was sequenced as part of the Darwin Tree of Life Project, a collaborative effort to sequence all named eukaryotic species in the Atlantic Archipelago of Britain and Ireland. Here we present a chromosomally complete genome sequence for *L. halterata*, based on the ilLobHalt1 specimen from Wytham Woods, Oxfordshire, UK.

### Genome sequence report

**Figure 1.**
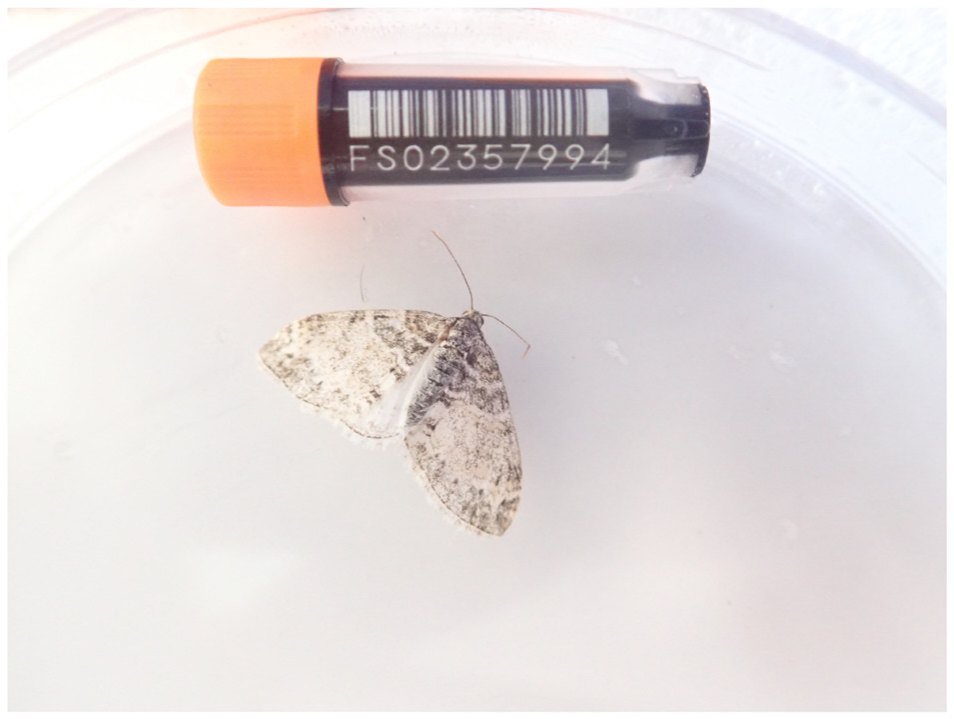
Photograph of the *Lobophora halterata* (ilLobHalt1) specimen used for genome sequencing.

The genome was sequenced from an individual female *L. halterata* ( Figure 1) collected from Wytham Woods, Berkshire, UK (51.77, –1.34). A total of 72-fold coverage in Pacific Biosciences single-molecule HiFi long reads was generated. Primary assembly contigs were scaffolded with chromosome conformation Hi-C data. Manual assembly curation corrected four missing or mis-joins, reducing the scaffold number by 10.53%.

**Figure 2.**
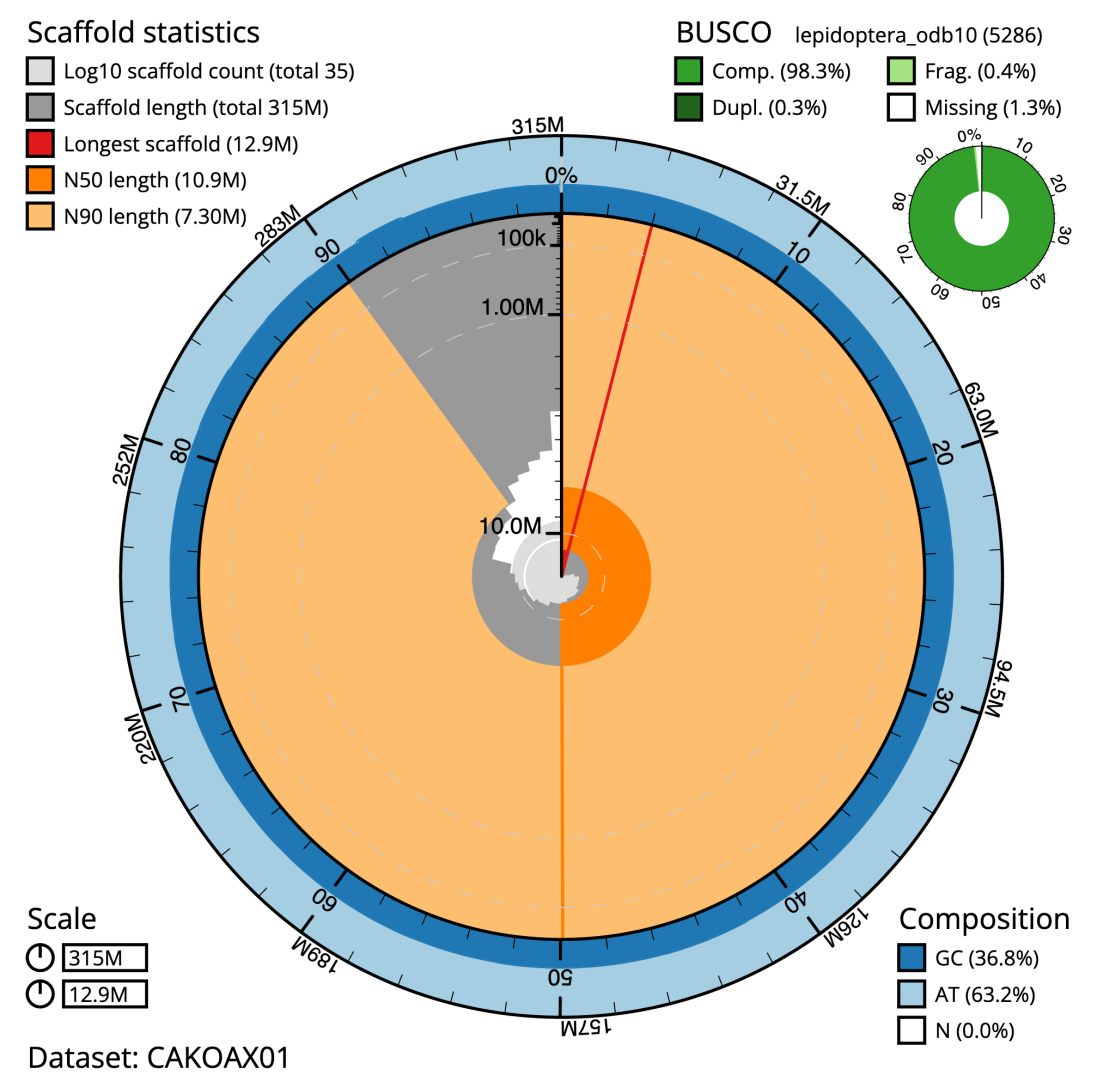
Genome assembly of *Lobophora halterata*, ilLobHalt1.1: metrics. The BlobToolKit Snailplot shows N50 metrics and BUSCO gene completeness. The main plot is divided into 1,000 size-ordered bins around the circumference with each bin representing 0.1% of the 314,876,238 bp assembly. The distribution of scaffold lengths is shown in dark grey with the plot radius scaled to the longest scaffold present in the assembly (12,870,039 bp, shown in red). Orange and pale-orange arcs show the N50 and N90 scaffold lengths (10,931,577 and 7,303,528 bp), respectively. The pale grey spiral shows the cumulative scaffold count on a log scale with white scale lines showing successive orders of magnitude. The blue and pale-blue area around the outside of the plot shows the distribution of GC, AT and N percentages in the same bins as the inner plot. A summary of complete, fragmented, duplicated and missing BUSCO genes in the lepidoptera_odb10 set is shown in the top right, An interactive version of this figure is available at https://blobtoolkit.genomehubs.org/view/ilLobHalt1.1/dataset/CAKOAX01/snail.

The final assembly has a total length of 314.9 Mb in 34 sequence scaffolds with a scaffold N50 of 10.9 Mb ( Table 1). The complete assembly sequence was assigned to 32 chromosomal-level scaffolds, representing 30 autosomes and the W and Z sex chromosomes. Chromosome-scale scaffolds confirmed by the Hi-C data are named in order of size. ( Figure 2– Figure 5; Table 2). The mitochondrial genome was also assembled. The assembly has a BUSCO v5.3.2 ( Manni *et al.*, 2021) completeness of 98.3% (single 98.0%, duplicated 0.3%), using the lepidoptera_odb10 reference set. Evaluation of the assembly shows a consensus quality value (QV) of 71.9 and *k*-mer completeness of 100%. While not fully phased, the assembly deposited is of one haplotype. Contigs corresponding to the second haplotype have also been deposited.

**Table 1.** Genome data for *Lobophora halterata*, ilLobHalt1.1.

Project accession data		
Assembly identifier	ilLobHalt1.1	
Species	*Lobophora halterata*	
Specimen	ilLobHalt1	
NCBI taxonomy ID	934876	
BioProject	PRJEB50743	
BioSample ID	SAMEA7520514	
Isolate information	female; thorax (PacBio sequencing), head (Hi-C)	
Assembly metrics *		*Benchmark*
Consensus quality (QV)	71.9	*≥ 50*
*k*-mer completeness	100%	*≥ 95%*
BUSCO **	C:98.3%[S:98.0%,D:0.3%], F:0.4%,M:1.3%,n:5286	*C ≥ 95%*
Percentage of assembly mapped to chromosomes	100%	*≥ 95%*
Sex chromosomes	Z and W sex chromosomes	*localised homologous pairs*
Organelles	Mitochondrial genome assembled	*complete single alleles*
Raw data accessions		
PacificBiosciences SEQUEL II	ERR8575379	
Hi-C Illumina	ERR8571665	
Genome assembly		
Assembly accession	GCA_945859715.1	
*Accession of alternate haplotype*	GCA_945859755.1	
Span (Mb)	314.9	
Number of contigs	38	
Contig N50 length (Mb)	10.9	
Number of scaffolds	34	
Scaffold N50 length (Mb)	10.9	
Longest scaffold (Mb)	12.9	

*Assembly metric benchmarks are adapted from column VGP-2020 of “Table 1: Proposed standards and metrics for defining genome assembly quality” from ( Rhie *et al.*, 2021).

**BUSCO scores based on the lepidoptera_odb10 BUSCO set using v5.3.2. C = complete [S = single copy, D = duplicated], F = fragmented, M = missing, n = number of orthologues in comparison. A full set of BUSCO scores is available at https://blobtoolkit.genomehubs.org/view/ilLobHalt1.1/dataset/CAKOAX01/busco.

**Figure 3.**
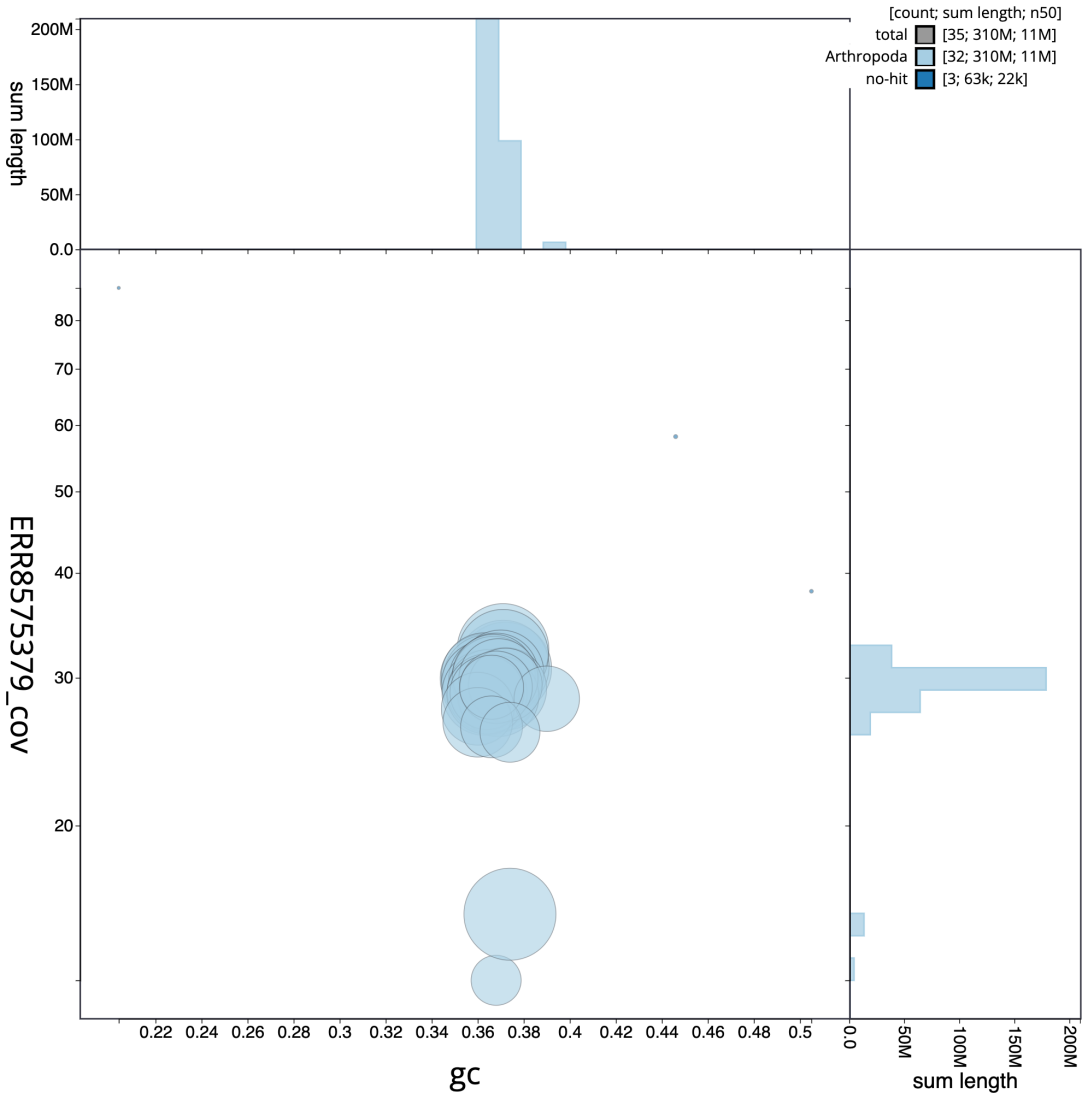
Genome assembly of *Lobophora halterata*, ilLobHalt1.1: GC coverage. BlobToolKit GC-coverage plot. Chromosomes are coloured by phylum. Circles are sized in proportion to scaffold length. Histograms show the distribution of scaffold length sum along each axis. An interactive version of this figure is available at https://blobtoolkit.genomehubs.org/view/ilLobHalt1.1/dataset/CAKOAX01/blob.

**Figure 4.**
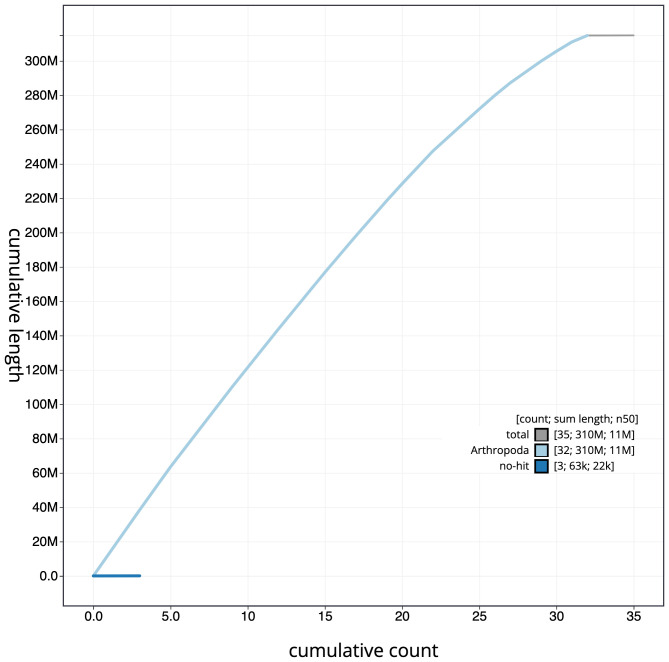
Genome assembly of *Lobophora halterata*, ilLobHalt1.1: cumulative sequence. BlobToolKit cumulative sequence plot. The grey line shows cumulative length for all scaffolds. Coloured lines show cumulative lengths of scaffolds assigned to each phylum using the buscogenes taxrule. An interactive version of this figure is available at https://blobtoolkit.genomehubs.org/view/ilLobHalt1.1/dataset/CAKOAX01/cumulative.

**Figure 5.**
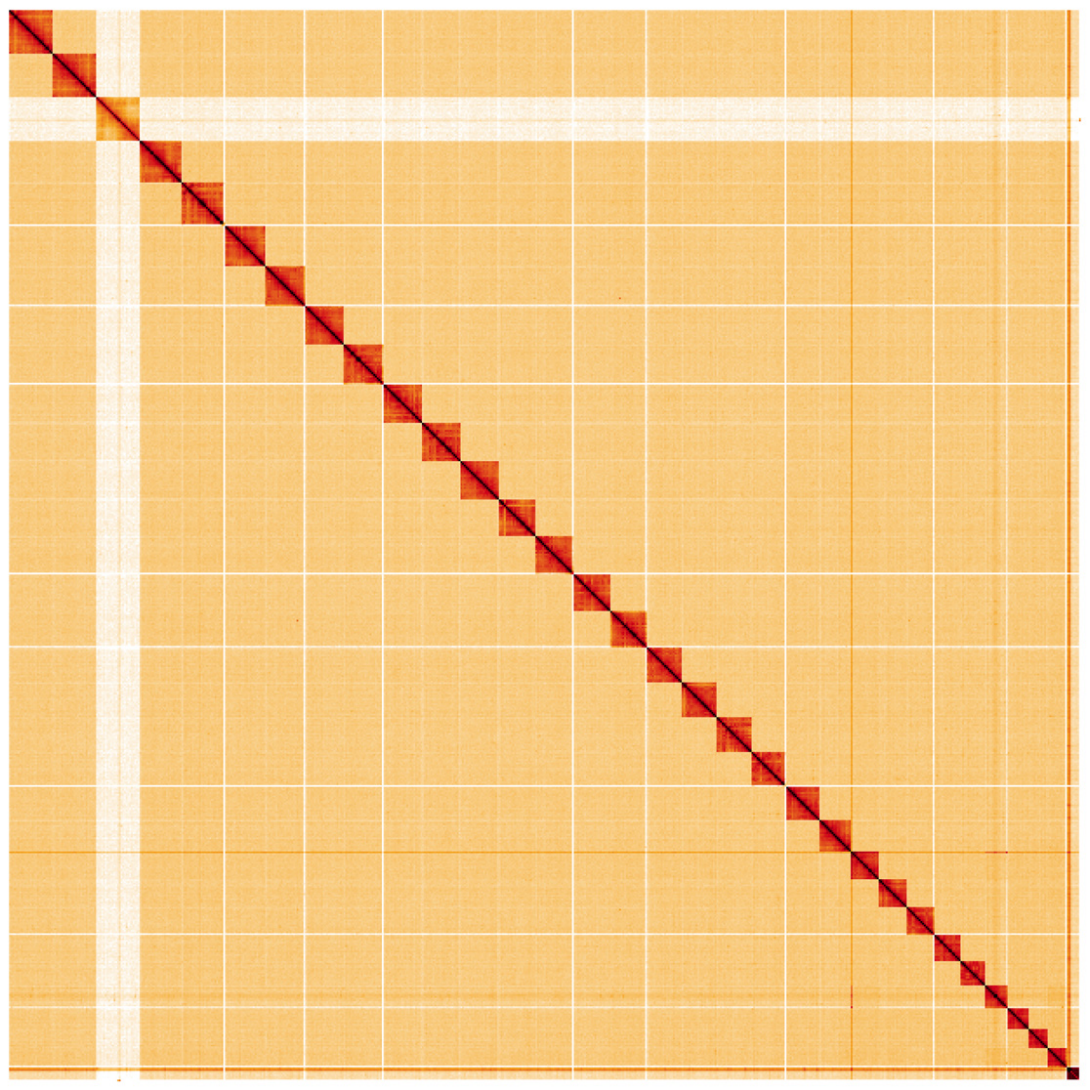
Genome assembly of *Lobophora halterata*, ilLobHalt1.1: Hi-C contact map. Hi-C contact map of the ilLobHalt1.1 assembly, visualised using HiGlass. Chromosomes are shown in order of size from left to right and top to bottom. An interactive version of this figure may be viewed at https://genome-note-higlass.tol.sanger.ac.uk/l/?d=T1L-6HBOROe3dhzQyJ_IRQ.

**Table 2.** Chromosomal pseudomolecules in the genome assembly of *Lobophora halterata*, ilLobHalt1.

INSDC accession	Chromosome	Size (Mb)	GC%
OW052001.1	1	12.87	37.2
OW052002.1	2	12.81	37.1
OW052004.1	3	12.7	37.1
OW052005.1	4	12.41	37.1
OW052006.1	5	11.92	36.3
OW052007.1	6	11.58	36.8
OW052008.1	7	11.57	36.7
OW052009.1	8	11.53	36.4
OW052010.1	9	11.28	36.3
OW052011.1	10	11.24	36.3
OW052012.1	11	11.24	37
OW052013.1	12	11.05	36.6
OW052014.1	13	10.93	36.8
OW052015.1	14	10.93	36.3
OW052016.1	15	10.61	36.7
OW052017.1	16	10.54	36.5
OW052018.1	17	10.3	36.7
OW052019.1	18	10.26	37.2
OW052020.1	19	9.87	36.9
OW052021.1	20	9.71	36.4
OW052022.1	21	9.41	36.9
OW052023.1	22	8.24	36.4
OW052024.1	23	8.19	37.2
OW052025.1	24	8	36
OW052026.1	25	7.92	36.8
OW052027.1	26	7.3	36
OW052028.1	27	6.47	39
OW052029.1	28	6.2	36.6
OW052030.1	29	5.77	36.6
OW052031.1	30	5.42	37.4
OW052032.1	W	3.78	36.8
OW052003.1	Z	12.77	37.4
OW052033.1	MT	0.02	20.5
-	-	0.05	47.3

## Methods

### Sample acquisition and nucleic acid extraction

A male *L. halterata* (ilLobHalt1) was collected using a light trap from Wytham Woods, Berkshire, UK (latitude 51.77, longitude –1.34) by Douglas Boyes (University of Oxford). The sample was identified by Douglas Boyes and snap-frozen on dry ice.

DNA was extracted at the Tree of Life laboratory, Wellcome Sanger Institute. The ilLobHalt1 sample was weighed and dissected on dry ice with tissue set aside for Hi-C sequencing. Abdomen tissue was cryogenically disrupted to a fine powder using a Covaris cryoPREP Automated Dry Pulveriser, receiving multiple impacts. High molecular weight (HMW) DNA was extracted using the Qiagen MagAttract HMW DNA extraction kit. HMW DNA was sheared into an average fragment size of 12–20 kb in a Megaruptor 3 system with speed setting 30. Sheared DNA was purified by solid-phase reversible immobilisation using AMPure PB beads with a 1.8X ratio of beads to sample to remove the shorter fragments and concentrate the DNA sample. The concentration of the sheared and purified DNA was assessed using a Nanodrop spectrophotometer and Qubit Fluorometer and Qubit dsDNA High Sensitivity Assay kit. Fragment size distribution was evaluated by running the sample on the FemtoPulse system.

### Sequencing

Pacific Biosciences HiFi circular consensus and 10X Genomics read cloud DNA sequencing libraries were constructed according to the manufacturers’ instructions. DNA sequencing was performed by the Scientific Operations core at the WSI on the Pacific Biosciences SEQUEL II (HiFi) instrument. Hi-C data were also generated from head/thorax tissue of ilLobHalt1 using the Arima v2 kit and sequenced on the Illumina HiSeq X Ten instrument.

### Genome assembly

Assembly was carried out with Hifiasm ( Cheng *et al.*, 2021) and haplotypic duplication was identified and removed with purge_dups ( Guan *et al.*, 2020). The assembly was scaffolded with Hi-C data ( Rao *et al.*, 2014) using YaHS ( Zhou *et al*., 2022). The assembly was checked for contamination as described previously ( Howe *et al.*, 2021). Manual curation was performed using HiGlass ( Kerpedjiev *et al.*, 2018) and Pretext ( Harry, 2022). The mitochondrial genome was assembled using MitoHiFi ( Uliano-Silva *et al.*, 2021), which performed annotation using MitoFinder ( Allio *et al.*, 2020). The genome was analysed and BUSCO scores generated within the BlobToolKit environment ( Challis *et al.*, 2020). Table 3 contains a list of all software tool versions used, where appropriate.

**Table 3.** Software tools and versions used.

Software tool	Version	Source
BlobToolKit	3.4.0	Challis *et al.*, 2020
Hifiasm	0.16.1-r375	Cheng *et al.*, 2021
HiGlass	1.11.6	Kerpedjiev *et al.*, 2018
MitoHiFi	2	Uliano-Silva *et al.*, 2021
PretextView	0.2	Harry, 2022
purge_dups	1.2.3	Guan *et al.*, 2020
YaHS	yahs-1.1.91eebc2	Zhou *et al*., 2022

### Ethics/compliance issues

The materials that have contributed to this genome note have been supplied by a Darwin Tree of Life Partner. The submission of materials by a Darwin Tree of Life Partner is subject to the Darwin Tree of Life Project Sampling Code of Practice. By agreeing with and signing up to the Sampling Code of Practice, the Darwin Tree of Life Partner agrees they will meet the legal and ethical requirements and standards set out within this document in respect of all samples acquired for, and supplied to, the Darwin Tree of Life Project. Each transfer of samples is further undertaken according to a Research Collaboration Agreement or Material Transfer Agreement entered into by the Darwin Tree of Life Partner, Genome Research Limited (operating as the Wellcome Sanger Institute), and in some circumstances other Darwin Tree of Life collaborators.

## Data Availability

European Nucleotide Archive: *Lobophora halterata* (the seraphim). Accession number PRJEB50743; https://identifiers.org/ena.embl/PRJEB50743 ( Wellcome Sanger Institute, 2022) The genome sequence is released openly for reuse. The *Lobophora halterata* genome sequencing initiative is part of the Darwin Tree of Life (DToL) project. All raw sequence data and the assembly have been deposited in INSDC databases *:* The genome will be annotated and presented through the Ensembl pipeline at the European Bioinformatics Institute. Raw data and assembly accession identifiers are reported in Table 1.

## Author information

Members of the University of Oxford and Wytham Woods Genome Acquisition Lab are listed here: https://doi.org/10.5281/zenodo.4789928.

Members of the Darwin Tree of Life Barcoding collective are listed here: https://doi.org/10.5281/zenodo.4893703.

Members of the Wellcome Sanger Institute Tree of Life programme are listed here: https://doi.org/10.5281/zenodo.4783585.

Members of Wellcome Sanger Institute Scientific Operations: DNA Pipelines collective are listed here: https://doi.org/10.5281/zenodo.4790455.

Members of the Tree of Life Core Informatics collective are listed here: https://doi.org/10.5281/zenodo.5013541.

Members of the Darwin Tree of Life Consortium are listed here: https://doi.org/10.5281/zenodo.4783558.

## References

[ref-1] Allio R Schomaker-Bastos A Romiguier J : MitoFinder: Efficient automated large-scale extraction of mitogenomic data in target enrichment phylogenomics. * Mol Ecol Resour.* 2020;20(4):892–905. 10.1111/1755-0998.13160 32243090 PMC7497042

[ref-2] Challis R Richards E Rajan J : BlobToolKit - interactive quality assessment of genome assemblies. * G3 (Bethesda).* 2020;10(4):1361–1374. 10.1534/g3.119.400908 32071071 PMC7144090

[ref-3] Cheng H Concepcion GT Feng X : Haplotype-resolved de novo assembly using phased assembly graphs with hifiasm. * Nat Methods.* 2021;18(2):170–175. 10.1038/s41592-020-01056-5 33526886 PMC7961889

[ref-4] GBIF Secretariat: Lobophora halterata (Hufnagel, 1767), GBIF Backbone Taxonomy. Checklist dataset. (Accessed: 4 December 2022).2021. Reference Source

[ref-5] Guan D McCarthy SA Wood J : Identifying and removing haplotypic duplication in primary genome assemblies. *Bioinformatics.* 2020;36(9):2896–2898. 10.1093/bioinformatics/btaa025 31971576 PMC7203741

[ref-6] Harry E : PretextView (Paired REad TEXTure Viewer): A desktop application for viewing pretext contact maps. (Accessed: 19 October 2022).2022. Reference Source

[ref-7] Hobby BM : A note on the hind-wings of a sawfly, leaf-hopper and moth. *Proceedings of the Royal Entomological Society of London. Series A General Entomology.* 2009;12(4–6):72–74. 10.1111/j.1365-3032.1937.tb00955.x

[ref-8] Holland P : The unusual hindwing of the Seraphim moth. *Bulletin of the Amateur Entomologists’ Society.* 2012;71:18–20. Reference Source

[ref-9] Howe K Chow W Collins J : Significantly improving the quality of genome assemblies through curation. *GigaScience.* Oxford University Press.2021;10(1):giaa153. 10.1093/gigascience/giaa153 33420778 PMC7794651

[ref-10] Kerpedjiev P Abdennur N Lekschas F : HiGlass: web-based visual exploration and analysis of genome interaction maps. *Genome Biol.* 2018;19(1):125. 10.1186/s13059-018-1486-1 30143029 PMC6109259

[ref-11] Maitland Emmet A : The Scientific Names of the British Lepidoptera.Colchester, UK: Harley Books.1991. Reference Source

[ref-12] Manni M Berkeley MR Seppey M : BUSCO Update: Novel and Streamlined Workflows along with Broader and Deeper Phylogenetic Coverage for Scoring of Eukaryotic, Prokaryotic, and Viral Genomes. *Mol Biol Evol.* 2021;38(10):4647–4654. 10.1093/molbev/msab199 34320186 PMC8476166

[ref-13] Rao SSP Huntley MH Durand NC : A 3D map of the human genome at kilobase resolution reveals principles of chromatin looping. *Cell.* 2014;159(7):1665–1680. 10.1016/j.cell.2014.11.021 25497547 PMC5635824

[ref-14] Rhie A McCarthy SA Fedrigo O : Towards complete and error-free genome assemblies of all vertebrate species. *Nature.* 2021;592(7856):737–746. 10.1038/s41586-021-03451-0 33911273 PMC8081667

[ref-15] Uliano-Silva M : MitoHiFi. 2021; (Accessed: 19 October 2022). Reference Source

[ref-16] Waring P Townsend M : Field Guide to the Moths of Great Britain and Ireland: Third Edition.Bloomsbury.2017. Reference Source

[ref-18] Wellcome Sanger Institute: The genome sequence of the Seraphim, *Lobophora halterata* (Hufnagel, 1767). European Nucleotide Archive.[dataset].2022; accession number PRJEB50743. 10.12688/wellcomeopenres.18713.1 PMC9975409 36874578

[ref-17] Zhou C McCarthy SA Durbin R : YaHS: yet another Hi-C scaffolding tool. *bioRxiv.* [Preprint].2022. 10.1101/2022.06.09.495093 PMC9848053 36525368

