# Supplementary material for: The genome sequence of the Seraphim, Lobophora halterata (Hufnagel, 1767)
Source: Wellcome Open Res. 2022 Dec 23;7:313. [Version 1] doi: 10.12688/wellcomeopenres.18713.1 (PMC9975409; doi:10.12688/wellcomeopenres.18713.1)
Supplement: Peer review history

# Peer review: Reviewer response for version 1

**DOI:** 10.21956/wellcomeopenres.20751.r53775

**Published:** 2023-01-16

**Authors:** Satoshi Yamamoto

**Affiliations:** 1 Institute for Agro-Environmental Sciences, NARO (NIAES), Tsukuba, Japan

This study presents a *de novo* genome assembly of *Lobophora halterata* (Lepidoptera; Geometridae) using a high coverage HiFi long read dataset. Scaffolding with Hi-C data subsequently yielded 32 chromosome-scale scaffolds. The assembly techniques employed conform to standard genome assembly methods, and the resulting chromosomal count is consistent with previous reports on chromosomes of geometrid species (for example, Suomalainen 1965 ^1^). As such, the assembly can be considered credible.

 However, the methodology section does not sufficiently detail the methods employed for quality control of raw reads, the options and parameters utilized in the assembly program, or the approach for identifying sex chromosomes.

 All data generated in this study have been deposited in public databases for accessibility. This study is deemed valuable given the relative scarcity of chromosome-scale genome assemblies for geometrid species.

Are sufficient details of methods and materials provided to allow replication by others?

Yes

Is the rationale for creating the dataset(s) clearly described?

Yes

Are the datasets clearly presented in a useable and accessible format?

Yes

Are the protocols appropriate and is the work technically sound?

Partly

Reviewer Expertise:

Evolutionary biology

I confirm that I have read this submission and believe that I have an appropriate level of expertise to confirm that it is of an acceptable scientific standard.

## References

[1] : On the chromosomes of the geometrid moth genus Cidaria. *Chromosoma* .1965;16(2) : 10.1007/BF00320947 166-184 10.1007/BF00320947

# Peer review: Reviewer response for version 1

**DOI:** 10.21956/wellcomeopenres.20751.r53776

**Published:** 2023-01-10

**Authors:** Yandong Ren

**Affiliations:** 1 College of Life Sciences, Shaanxi Normal University, Xi'an, China

This manuscript provided us a high-quality *Lobophora halterata* genome. The protocols are well described and the datasets are clearly presented. However, the details of methods and materials are not sufficient. How was the library constructed? What's the version of the software used in this manuscript? More detail should be added in the revised manuscript. Besides, I know this manuscript is a data note paper, the following analysis still need added:

1. The authors performed k-mer analysis. So what software was used in this analysis. The k-mer results should also be showed in the results part.
2. How about the N50 and the mean length of the raw reads.
3. How about the genome quality of other Geometridae species? The authors should added a table with the genome quality of all the published Geometridae genome.

Are sufficient details of methods and materials provided to allow replication by others?

Partly

Is the rationale for creating the dataset(s) clearly described?

Yes

Are the datasets clearly presented in a useable and accessible format?

Yes

Are the protocols appropriate and is the work technically sound?

Yes

Reviewer Expertise:

Genomics

I confirm that I have read this submission and believe that I have an appropriate level of expertise to confirm that it is of an acceptable scientific standard.
